# Recognition of the D.D.S. Degree by the American Medical Association

**Published:** 1903-07

**Authors:** 


### RECOGNITION OF THE D.D.S. DEGREE BY THE AMERICAN MEDICAL ASSOCIATION.

UNDER the proper heading will be found a communication from Dr. Eugene S. Talbot, secretary of the Section on Stomatology, American Medical Association, announcing the change of attitude of the latter towards those holding the degree of Doctor of Dental Surgery (D.D.S.) or its equivalent. This alteration in the by-laws permitting the holder of the D.D.S. degree to become a member of the Section is indicative of a marked advance in the feeling of the medical profession towards the dental. Whether dentistry should be profoundly grateful for this at this late stage of progress is a question that must be individually answered. In the opinion of the writer the change should have been made long ago. The better class of dentists not burdened with the M.D. degree have taken but a very mild interest in the action of the American Medical Association. The general feeling has been that it would be much better to continue to occupy an independent position rather than one of sufferance in a body giving them only partial recognition. The result of this feeling has been injurious to the Section, and through paucity of numbers has generated weakness, for while numbers are not important in a scientific sense, they do bring an invigorating influence. Whether this change in the attitude of the medical profession towards dentistry will help to give renewed life to the dental section or not, it will largely eradicate the feeling of prejudice that has existed against this body. It must, however, be borne in mind by those at present in membership that any large additions to its body can hardly be expected so long as the Section refuses to receive papers upon the technique of dentistry. To accept part of a profession and not its whole is not only an absurdity, but the D.D.S. naturally and very justifiedly resents it. He will, or, at least, should, say, Accept me as I am, or not at all. The mechanical part of my profession is of more importance to me than the diagnosis and therapeutics of the medical profession. Any other position is lowering of self-respect.

The claim set up by Dr. Talbot, that the "influence [of this Section] has been felt in universities in the advancement of their years of study, preliminary education, groundwork in medical principles, in the passing of the Army Medical Bill, and in the establishment of the Army Dental Corps," must be recognized as being equally true with that exercised by all dental bodies and by individuals.

No one organization can claim this honor; indeed, it is a question whether any one can assume to have been the medium of progress. This comes through natural development. Medical science to-day is not the medical science of thirty years ago, and dental science has undergone almost a total change in a similar period. The religious conceptions are radically altered from the views held fifty years back. These changes are all the result of scientific progress and mental illumination, peculiar to no era or to no class, but characteristic of progressive civilization.

